# Supplementary material for: Theoretical Insight into the Reaction Mechanism and Kinetics for the Criegee Intermediate of anti-PhCHOO with SO2
Source: Molecules. 2020 Jul 3;25(13):3041. doi: 10.3390/molecules25133041 (PMC7412395; doi:10.3390/molecules25133041)
Supplement: Supplementary file 1 [file molecules-25-03041-s001.pdf]

Supplementary Materials

Theoretical Insight into the Reaction Mechanism and Kinetics for the Criegee Intermediate of *anti*-PhCHOO with SO_2

Benni Du, Weichao Zhang*

School of Chemistry and Materials Science, Jiangsu Normal University, Xuzhou, Jiangsu 221116, People's Republic of China

* Corresponding author. Tel: +86-516-83403165; fax: +86 516 83403164

E-mail address: zwc@jsnu.edu.cn

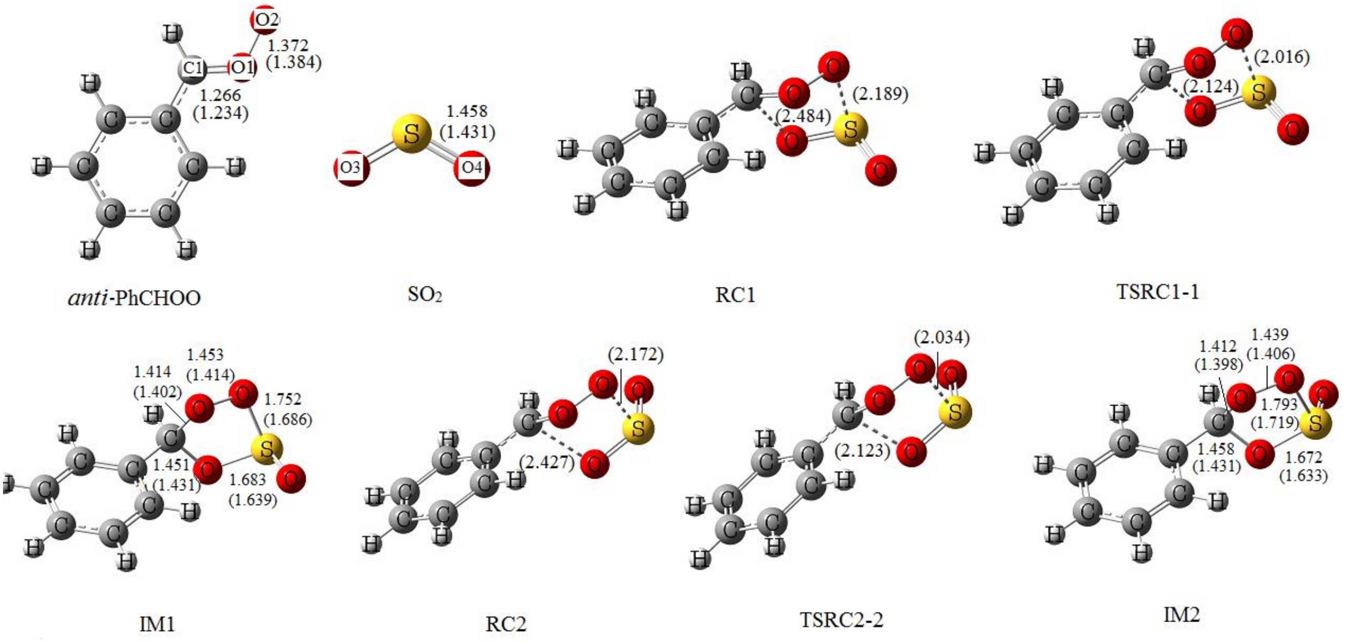

Figure S1 Optimized geometries of species including reactants, reactant complexes, transition states and intermediates involved in the reaction of *anti*-PhCHOO+SO_2 at the UB3LYP/6-311++G(d,p) level. The values in parentheses are from UBH&HLYP/6-311++G(d,p) level. Bond lengths are given in angstrom.

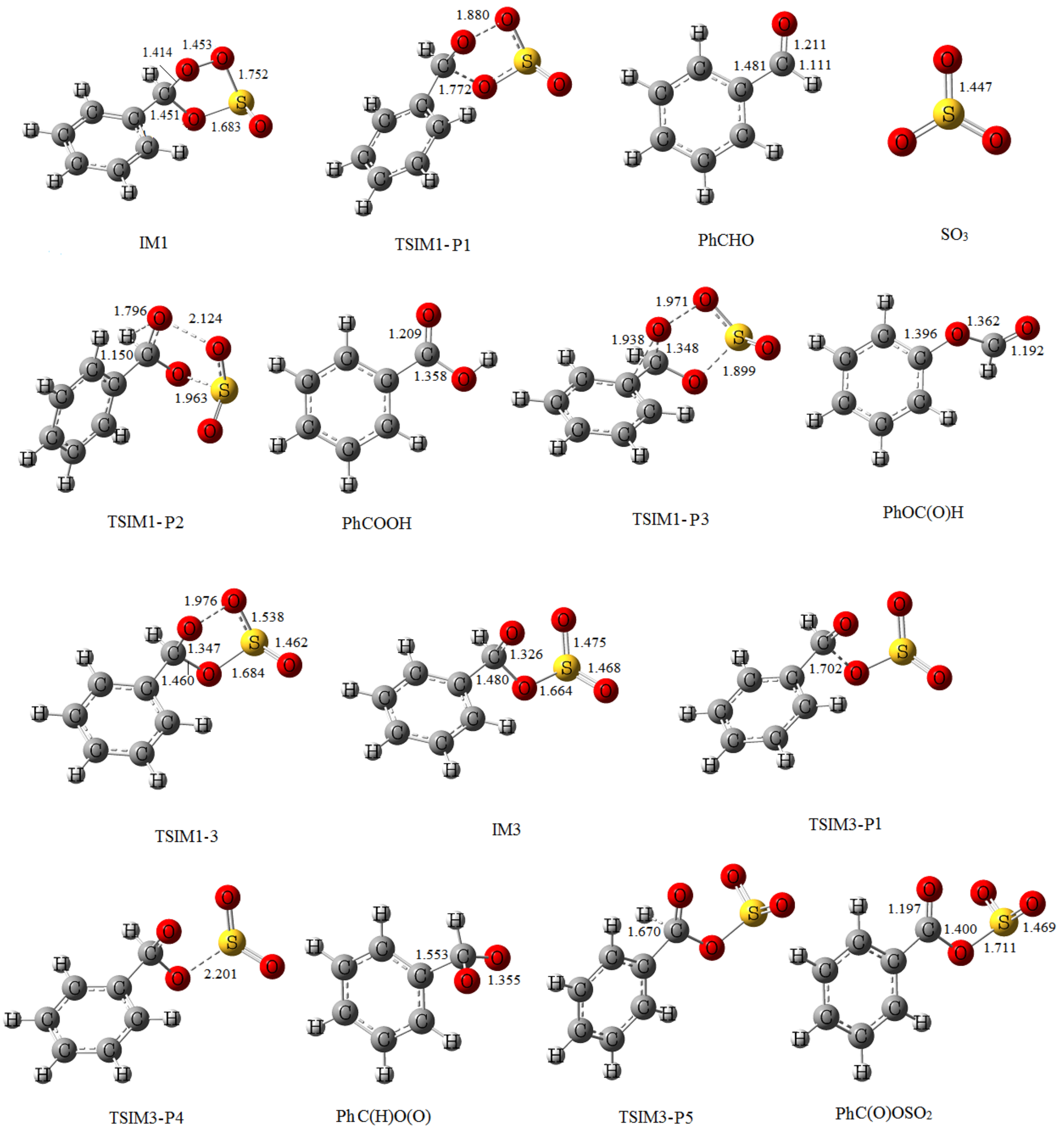

Figure S2 Optimized geometries of various species involved in the subsequent reaction pathways of IM1 at the UB3LYP/6-311++G(d,p) level.

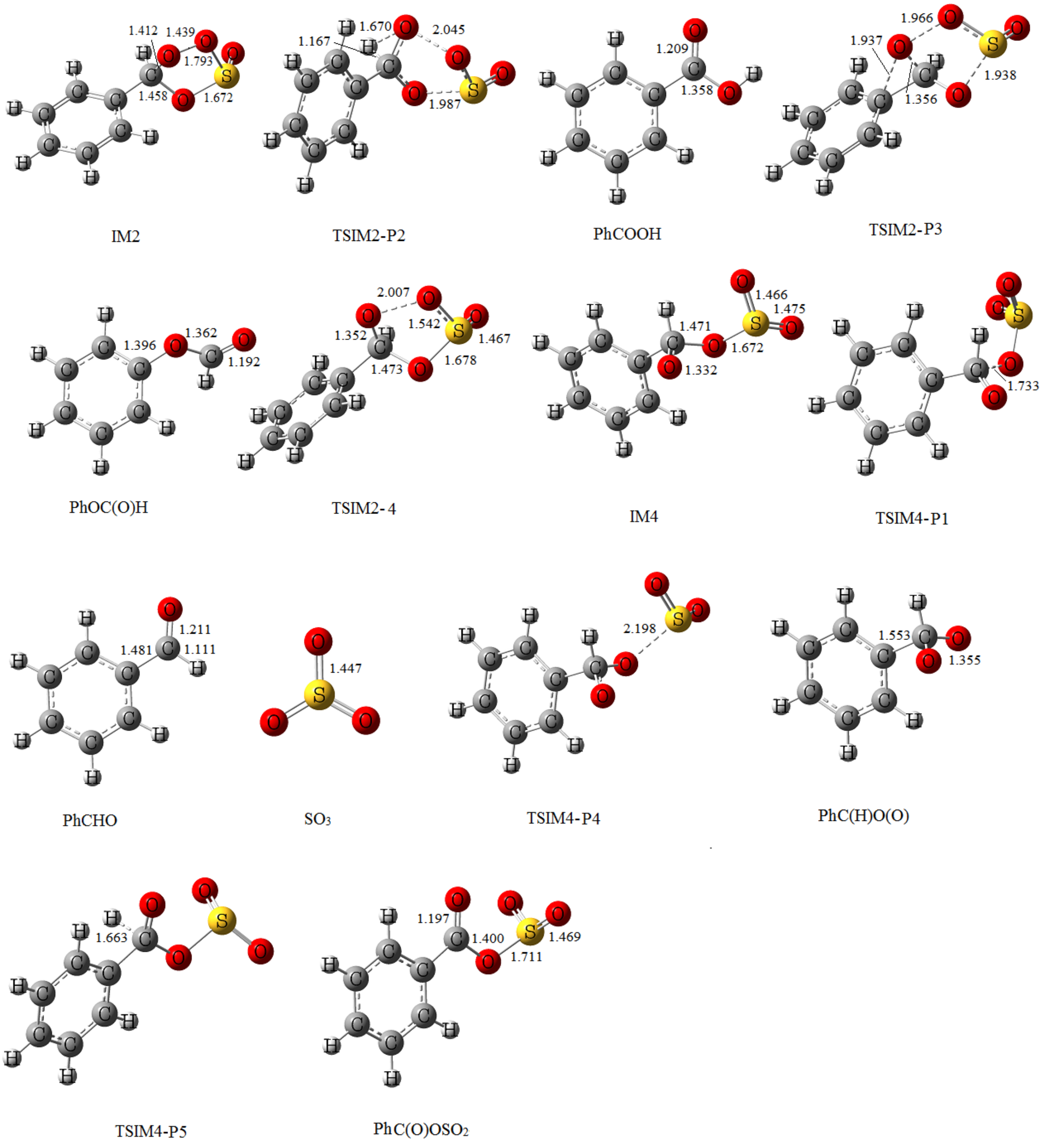

Figure S3 Optimized geometries of various species involved in the subsequent reaction pathways of IM2 at the UB3LYP/6-311++G(d,p) level.

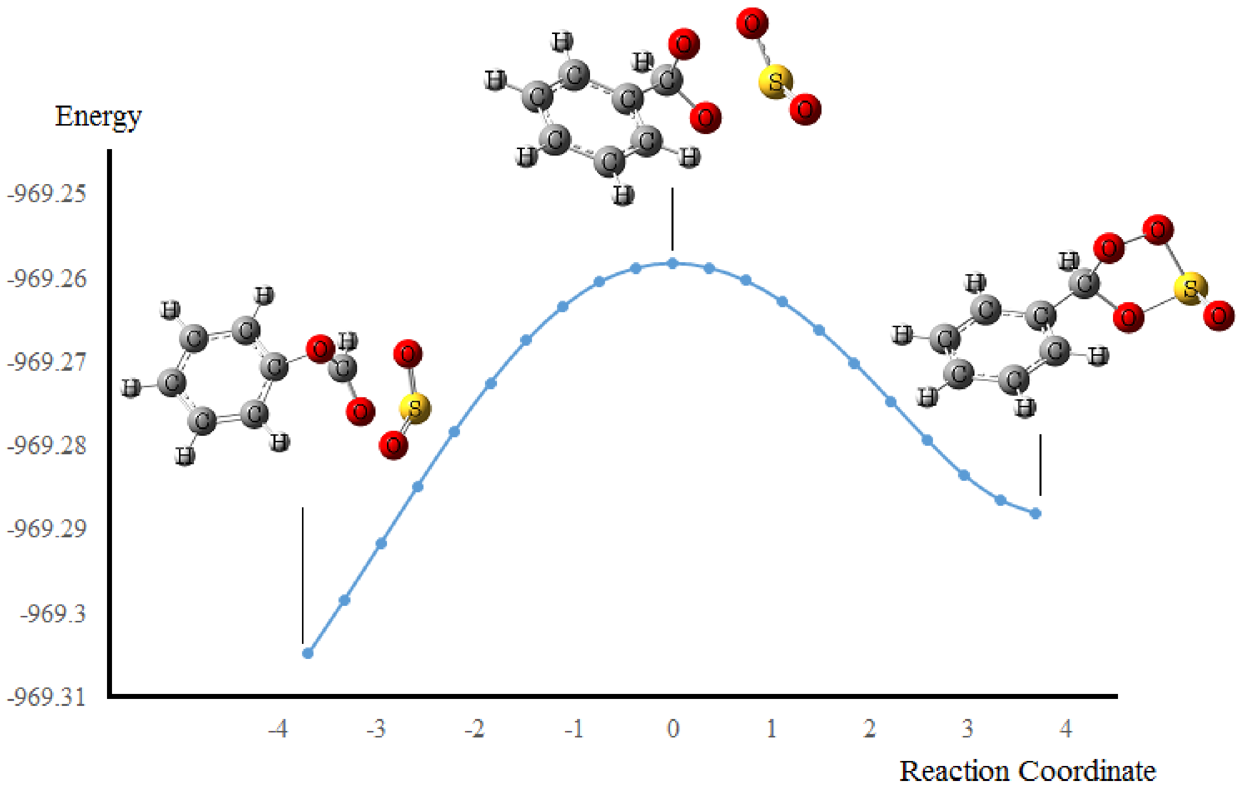

Figure S4 The intrinsic reaction coordinate of TSIM1-P3.

Table S1 The total energies (hartree) obtained at the UCCSD(T)//UB3LYP and UCCSD//UB3LYP levels of theory together with the %TAE(T) of various species in the reaction of *anti*-PhCHOO+SO_2

Species	E(UCCSD(T))[a]	E(UCCSD)[b]	%TAE(T)
anti-PhCHOO	-419.6801484	-419.6116184	0.016
	(-419.6810256)	(–419.6128284)	(0.016)
SO_2	-547.8045786	-547.7769942	0.005
	(-547.8026436)	(–547.7767351)	(0.005)
RC1	(-967.5109488)	(–967.4154152)	(0.010)
TSRC1-I	(-967.5105243)	(–967.4143004)	(0.010)
IM1	-967.5449843	-967.4503023	0.010
	(-967.539798)	(–967.4489013)	(0.009)
RC2	(-967.5129371)	(–967.4169875)	(0.010)
TSRC2-2	(-967.5131491)	(–967.4164146)	(0.010)
IM2	-967.5439359	-967.4488583	0.010
	(-967.5385981)	(–967.4473619)	(0.009)
TS-IM1-P1	-967.4999876	-967.3976815	0.011
PhCHO	-344.756284	-344.6987848	0.017
SO_3	-622.8300556	-622.7947814	0.006
TS-IM1-P2	-967.4889016	-967.3901906	0.010
PhCOOH	-419.8725338	-419.8077707	0.015
TS-IM1-P3	-967.4944088	-967.3923751	0.011
PhOC(O)H	-419.8433275	-419.7782968	0.015
TS-IM1-3	-967.505543	-967.4104843	0.010
IM3	-967.5193193	-967.4291302	0.009
TS-IM3-P1	-967.5058978	-967.4141757	0.009
PhCHO	-344.756284	-344.6987848	0.017
TS-IM3-P4	-967.5007049	-967.4094430	0.009
PhCHOO	-419.7010942	-419.6409464	0.014
TS-IM3-P5	-967.4863683	-967.3918089	0.010
PhC(O)OSO2-1	-967.004954	-966.9109814	0.010
H	-0.499818	-0.4998179	0.000
TS-IM2-P2	-967.4951956	-967.3935992	0.011
TS-IM2-P3	-967.4860589	-967.3838216	0.011
TS-IM2-4	-967.498527	-967.4041150	0.010
IM4	-967.5204819	-967.4305090	0.009
TS-IM4-P1	-967.5056254	-967.4139643	0.009
TS-IM4-P4	-967.5022709	-967.4115855	0.009
TS-IM4-P5	-967.4889554	-967.3943054	0.010

[a] The values in parentheses are calculated at the UCCSD(T)/6-311++G(d,p)//UBH&HLYP/6-311++G(d,p) levels of theory.

[b] The values in parentheses are calculated at the UCCSD/6-311++G(d,p)//UBH&HLYP/6-311++G(d,p) levels of theory.

Table S2 The ZPE (hartree), total energies (E, hartree) and relative energies (ΔE, Δ(E+ZPE), kJ/mol) without and with ZPE corrections of various species calculated at the UCCSD(T)//UB3LYP levels of theory in the reaction of *anti*-PhCHOO+SO_2

Species	ZPE[a]	E(UCCSD(T))[a]	ΔE	E(UCCSD(T) +ZPE)[a]	Δ(E+ZPE) [a]
anti-PhCHOO+SO_2	0.119314	-967.4847270	0.00	-967.3691356	0.00
	(0.124066)	(-967.4836690)	(0.00)	(-967.3678536)	(0.00)
RC1	(0.127226)	(-967.5109488)	(-71.62)	(-967.3921833)	(-63.88)
TSRC1-I	(0.127437)	(-967.5105243)	(-70.51)	(-967.3915619)	(-62.25)
IM1	0.123873	-967.5449843	-158.21	-967.4249761	-146.61
	(0.129811)	(-967.5397980)	(-147.37)	(-967.4186194)	(-133.29)
RC2	(0.127293)	(-967.5129371)	(-76.84)	(-967.3941091)	(-68.93)
TSRC2-2	(0.127459)	(-967.5131491)	(-77.40)	(-967.3941661)	(-69.08)
IM2	0.123904	-967.5439359	-155.45	-967.4238977	-143.78
	(0.129710)	(-967.5385981)	(-144.22)	(-967.4175138)	(-130.38)
TS-IM1-P1	0.121216	-967.4999876	-40.07	-967.4238977	-35.23
PhCHO+SO_3(P1)	0.120992	-967.5863396	-266.78	-622.8187885	-262.52
TS-IM1-P2	0.117864	-967.4889016	-10.96	-967.374715	-14.65
PhCOOH+ SO_2(P2)	0.12186	-967.6771124	-505.11	-967.5590544	-498.63
TS-IM1-P3	0.121028	-967.4944088	-25.42	-967.3771569	-21.06
PhOC(O)H+SO_2 (P3)	0.120307	-967.6479060	-428.43	-967.5313527	-425.90
TS-IM1-3	0.121199	-967.5055430	-54.65	-967.3881254	-49.86
IM3	0.121157	-967.5193193	-90.82	-967.4019424	-86.13
TS-IM3-P1	0.119894	-967.5058978	-55.58	-967.3897445	-54.11
TS-IM3-P4	0.118298	-967.5007049	-41.95	-967.3860978	-44.53
PhC(H)O(O)+ SO_2 (P4)	0.116565	-967.5056730	-54.99	-967.3927446	-61.99
TS-IM3-P5	0.113197	-967.4863683	-4.31	-967.376703	-19.87
PhC(O)OSO_2+H(P5)	0.111554	-967.5047720	-52.63	-967.3966984	-72.37
TS-IM2-P2	0.118144	-967.4951956	-27.49	-967.3807377	-30.46
TS-IM2-P3	0.120903	-967.4860589	-3.50	-967.3689281	0.54
TS-IM2-4	0.120822	-967.4985270	-36.23	-967.3814746	-32.40
IM4	0.121303	-967.5204819	-93.87	-967.4029636	-88.82
TS-IM4-P1	0.118912	-967.5056254	-54.87	-967.3904235	-55.89
TS-IM4-P4	0.118047	-967.5022709	-46.06	-967.387907	-49.28
TS-IM4-P5	0.113276	-967.4889554	-11.10	-967.3792136	-26.46

[a] The values in parentheses are calculated at the UCCSD(T) // UBH&HLYP levels of theory.

Cartesian coordinates for all optimized structures at the UB3LYP/6-311++G(d,p) level of theory

***anti*-PhCHOO**

C	-1.43401500	0.53657000	-0.00013800
H	-1.81069700	1.55735300	-0.00012300
O	-2.30061900	-0.38682000	-0.00000400
O	-3.62644600	-0.03452600	0.00002300
C	-0.02823700	0.22684700	-0.00005700
C	0.88831400	1.29409200	-0.00000800
C	0.45187000	-1.09808700	-0.00001700
C	2.25510800	1.04339700	0.00005500
H	0.52217400	2.31494800	-0.00003200
C	1.81696400	-1.33708500	0.00004400
H	-0.25228100	-1.92060600	-0.00005500
C	2.72173400	-0.27041800	0.00008300
H	2.95553800	1.87001000	0.00008300
H	2.18365000	-2.35676500	0.00005900
H	3.78771300	-0.46606500	0.00013500

SO_2

S	0.00000000	0.00000000	0.37171400
O	0.00000000	1.25449300	-0.37171400
O	0.00000000	-1.25449300	-0.37171400

IM1

C	-0.28886300	-0.85477600	-0.14118300
H	-0.34475000	-1.89005500	-0.48948700
O	-0.87500500	-0.74002100	1.14058500
O	-2.28487200	-0.96444000	0.87241600
C	1.11303200	-0.33044100	-0.08207700
C	2.18022200	-1.19715000	-0.31803200
C	1.35326100	1.01195400	0.22550700
C	3.48990600	-0.72591100	-0.24515900
H	1.98982200	-2.23790900	-0.55759600
C	2.66091800	1.47852400	0.29767500
H	0.51882900	1.68074100	0.39818200
C	3.72944600	0.61117200	0.06261500
H	4.31790600	-1.40020400	-0.43018400
H	2.84884300	2.51924400	0.53478700
H	4.74754000	0.97986100	0.11761100
S	-2.68222100	0.14340500	-0.42495000
O	-1.11832300	-0.04699000	-1.01635200
O	-2.79557300	1.51315200	0.05707800

IM2

C	0.32126200	0.42508900	0.25714800
H	0.59502000	1.47719100	0.36839000
O	0.77968800	-0.32047200	1.36473600
O	2.21175900	-0.21525000	1.27853100
C	-1.14577700	0.20880000	0.07364000
C	-2.01075500	1.30311000	0.10857900
C	-1.65397600	-1.07979400	-0.11557600
C	-3.38234100	1.11308600	-0.04624300
H	-1.61375700	2.30162000	0.25632600
C	-3.02341800	-1.26639400	-0.26835500
H	-0.97741000	-1.92471400	-0.14465200
C	-3.88815700	-0.17129400	-0.23351900
H	-4.05199800	1.96468500	-0.02022300
H	-3.41838000	-2.26500000	-0.41481700
H	-4.95520200	-0.32069800	-0.35397500
S	2.67478300	-0.36111800	-0.44760200
O	1.07211500	-0.12965900	-0.86329400
O	3.47695800	0.83403000	-0.67790500

TSIM1-P1

C	-0.23199100	-0.95328000	0.38409200
H	-0.34223300	-2.00464100	0.10915400
O	-0.86963800	-0.52652800	1.42836400
O	-2.62998500	-0.69683100	0.79195600
C	1.13521300	-0.37913800	0.18189900
C	2.11127400	-1.18777900	-0.40173400
C	1.44400400	0.92233900	0.58861700
C	3.40057400	-0.69218000	-0.58625700
H	1.86566800	-2.19687300	-0.71383900
C	2.73076700	1.41097500	0.39960900
H	0.67842500	1.53797300	1.04369500
C	3.71004500	0.60563200	-0.18637000
H	4.15849000	-1.31864000	-1.04189400
H	2.97289800	2.42072100	0.70992800
H	4.71275800	0.99161900	-0.33036500
S	-2.55975500	0.12622900	-0.51636200
O	-1.13992600	-0.33055800	-1.00468600
O	-2.72160700	1.57776000	-0.43988500

PhCHO

C	-1.99213400	0.46354300	0.00008500
H	-2.27076200	1.53895000	0.00069100
O	-2.84858500	-0.39245200	-0.00027900

C	-0.53393900	0.20645100	0.00013200
C	0.35481700	1.28686700	0.00009700
C	-0.03820300	-1.10459100	0.00009600
C	1.72935100	1.06369300	-0.00015400
H	-0.03477600	2.30032300	0.00026700
C	1.33257600	-1.32592800	0.00010700
H	-0.74265600	-1.92808200	0.00010800
C	2.21652500	-0.24249600	-0.00011900
H	2.41747900	1.90109200	-0.00033300
H	1.71906600	-2.33872500	0.00028500
H	3.28637900	-0.41917900	-0.00024500

SO_3

S	0.00000000	0.00043300	0.00000000
O	-0.00257400	-1.44669900	0.00000000
O	1.25483700	0.72077600	0.00000000
O	-1.25226300	0.72505800	0.00000000

TSIM1-P2

C	-0.53104900	0.89533200	0.85818700
H	-0.31929900	1.48757800	1.82108100
O	-1.08291200	1.92240500	0.25502300
O	-2.28377500	0.63826700	-0.93618100
C	0.82989100	0.40817400	0.39872100
C	1.57016500	1.17194500	-0.51110300
C	1.36640500	-0.76990100	0.93322300
C	2.82174300	0.72914100	-0.92119600
H	1.14707800	2.08784600	-0.90414300
C	2.62147300	-1.20412500	0.52217300
H	0.78836600	-1.34508300	1.64534100
C	3.34955900	-0.45639400	-0.40349700
H	3.38694700	1.30462500	-1.64502400
H	3.03154000	-2.12427200	0.92145100
H	4.32931300	-0.79505400	-0.72097300
S	-2.24201400	-0.73076800	-0.29437000
O	-1.39713200	-0.04724200	1.34009000
O	-1.31878800	-1.70947900	-0.86729000

PhCOOH

C	-1.83860300	-1.23431200	-0.00002500
C	-0.44719400	-1.20008100	-0.00001600
C	0.21961000	0.03102500	0.00000400
C	-0.51533700	1.22234500	0.00001600
C	-1.90496400	1.18271600	0.00000800

C	-2.56768100	-0.04542400	-0.00001300
H	-2.35438900	-2.18766100	-0.00004200
H	0.12448000	-2.11877500	-0.00002500
H	0.01854400	2.16457500	0.00003200
H	-2.47210000	2.10639200	0.00001900
H	-3.65159900	-0.07564600	-0.00001900
C	1.70306000	0.12517900	0.00001300
O	2.33406400	1.15659900	0.00001100
O	2.31285100	-1.08875500	0.00000300
H	3.26640000	-0.92033300	-0.00000100

TSIM1-P3

C	0.33166800	-0.77117200	0.54068200
H	0.31259400	-1.74150700	1.05286300
O	0.43393300	-0.83599500	-0.80184000
O	2.39268100	-1.04701700	-0.72769500
C	-1.12729800	-0.27754600	0.20103200
C	-2.15592700	-1.22508200	0.11432700
C	-1.37460500	1.09249900	0.03312700
C	-3.46178100	-0.78501600	-0.05445100
H	-1.92826200	-2.28127500	0.19119700
C	-2.68352400	1.51498300	-0.13904100
H	-0.54873900	1.79058300	0.07044200
C	-3.72469200	0.58109300	-0.18191300
H	-4.27208900	-1.50273700	-0.09832500
H	-2.89763800	2.57171100	-0.24495900
H	-4.74455000	0.92011200	-0.32342200
S	2.79525700	0.11954600	0.19167800
O	1.13969400	0.14175200	1.12159400
O	2.85013200	1.43523700	-0.44171300

PhOC(O)H

C	2.33466700	0.16137200	0.35348000
H	1.87276000	0.68956500	1.20356100
O	1.41626100	-0.41549500	-0.47061700
C	0.06320500	-0.17779700	-0.22473900
C	-0.76495400	-1.28066800	-0.04394800
C	-0.44517100	1.11789000	-0.21950900
C	-2.12800500	-1.07808400	0.15640800
H	-0.33612800	-2.27498700	-0.06572800
C	-1.81073100	1.30817100	-0.00794700
H	0.21075200	1.96127600	-0.40101800
C	-2.65284200	0.21445700	0.17992400
H	-2.78018100	-1.93235600	0.29646400

H	-2.21432000	2.31404900	-0.00294400
H	-3.71398400	0.36714200	0.33738800
O	3.50675000	0.07590400	0.15440000

TSIM1-3

C	-0.28235200	-0.82770300	-0.17437900
O	-0.67004800	-0.87158100	1.11511600
O	-2.57493900	-1.02937100	0.61314900
O	-1.10187700	0.11240100	-0.93252000
S	-2.68532400	0.15539500	-0.36172000
O	-2.92639200	1.49049000	0.18301700
H	-0.33714400	-1.79661000	-0.68207100
C	1.15046400	-0.30269000	-0.10804300
C	2.20148400	-1.20479200	-0.27938700
C	1.40218300	1.04106000	0.18105700
C	3.51535500	-0.75121600	-0.19714600
H	1.99516200	-2.24898400	-0.48493400
C	2.71732200	1.48517400	0.26131300
H	0.57563700	1.72577500	0.32036100
C	3.77339300	0.59202800	0.07319400
H	4.33439300	-1.44528700	-0.34505100
H	2.91919600	2.52892100	0.47158100
H	4.79689500	0.94318500	0.13787900

IM3

C	-0.22838600	-0.72134100	0.16125500
O	-0.42577800	-0.81351100	1.46918300
O	-3.01578800	-1.13760800	0.19323700
O	-1.04840600	0.32757100	-0.48608400
S	-2.70221100	0.14625300	-0.46261700
O	-3.27161500	1.41491400	0.00863300
H	-0.41677300	-1.66581200	-0.36436000
C	1.22613400	-0.27673800	0.02103500
C	2.19438400	-1.20261100	-0.37455200
C	1.58531500	1.03222800	0.35880700
C	3.52284700	-0.80369500	-0.47369300
H	1.90962000	-2.22064600	-0.61445600
C	2.91714400	1.42198400	0.25655200
H	0.82433900	1.73336100	0.67554400
C	3.88493800	0.50730400	-0.15721500
H	4.27502200	-1.51271700	-0.79921800
H	3.19968900	2.43827900	0.50472600
H	4.92191300	0.81377600	-0.23325900

TSIM3-P1

C	-0.19413500	-0.83808600	0.44750300
O	-0.66313000	-0.52961000	1.59230800
O	-3.01219100	-0.91409000	0.45786500
O	-1.05014500	-0.01091100	-0.76841000
S	-2.60383500	0.11895200	-0.51417000
O	-3.03497300	1.51541600	-0.37351100
H	-0.36341600	-1.86338900	0.09825700
C	1.21197700	-0.33618500	0.18814300
C	2.11634200	-1.18490900	-0.44902900
C	1.60227200	0.93611300	0.61481800
C	3.42010100	-0.74980400	-0.67816500
H	1.80535100	-2.17173400	-0.77227800
C	2.90451300	1.36195700	0.38067100
H	0.88899100	1.58025600	1.11295000
C	3.81385700	0.52093300	-0.26342100
H	4.12373600	-1.40250700	-1.18150600
H	3.21211200	2.34997500	0.70259900
H	4.82853400	0.85761400	-0.44243400

TSIM3-P4

O	-0.39808400	-0.58624200	1.59012600
C	-0.13219700	-0.69483700	0.27757100
H	-0.24738700	-1.74372500	-0.05005500
O	-3.26912800	-0.93858100	0.53471700
O	-0.78407200	0.16622500	-0.58800300
S	-2.97997100	0.04841000	-0.50694800
O	-3.45777400	1.41614300	-0.34022600
C	1.35384100	-0.28777900	0.09159500
C	2.26303000	-1.17750000	-0.48311900
C	1.75494700	0.98898400	0.50221400
C	3.58916900	-0.78761300	-0.63651700
H	1.94062600	-2.16121400	-0.80237300
C	3.08477500	1.36690000	0.34293700
H	1.03251600	1.66778800	0.93593700
C	4.00051000	0.48243200	-0.22465000
H	4.30266700	-1.47411900	-1.07737400
H	3.40395700	2.35232200	0.66130000
H	5.03516200	0.78050500	-0.34936400

PhC(H)O(O)

C	1.79629900	0.35773800	0.00001000
O	2.22236000	-0.18594500	1.16524600
O	2.22254300	-0.18611200	-1.16507900

H	2.01293700	1.44599600	-0.00008900
C	0.25325100	0.18233600	-0.00001300
C	-0.59340300	1.29198800	0.00002700
C	-0.26522700	-1.11730700	-0.00006400
C	-1.97065700	1.09686600	0.00000400
H	-0.18287700	2.29452200	0.00007000
C	-1.64565000	-1.29957700	-0.00008800
H	0.40672000	-1.96559900	-0.00008600
C	-2.49738100	-0.19662400	-0.00005400
H	-2.63399900	1.95411600	0.00003000
H	-2.05379900	-2.30354000	-0.00013100
H	-3.57160100	-0.34156100	-0.00007100

TSIM3-P5

C	-0.23000500	0.50796700	0.13325700
O	-0.70344900	1.61957700	0.04805300
O	-3.08411800	0.03936500	1.25608600
O	-1.02817500	-0.65390700	-0.09626300
S	-2.71343300	-0.49137000	-0.06571100
O	-3.13595100	0.16353900	-1.31217000
H	-0.15533000	0.35433600	1.79479300
C	1.22471000	0.15680700	0.06239500
C	2.13791600	1.21645200	-0.01810400
C	1.67866300	-1.16812300	0.07100000
C	3.49857800	0.94746700	-0.09761100
H	1.76672700	2.23354700	-0.02362700
C	3.04269900	-1.42644400	-0.00831700
H	0.96782000	-1.98057700	0.13626700
C	3.95281500	-0.37248100	-0.09205800
H	4.20554500	1.76589200	-0.16653000
H	3.39621300	-2.45078900	-0.00546000
H	5.01524700	-0.57894900	-0.15310300

PhC(O)OSO$_2$

C	0.22872400	0.49183500	-0.00016300
O	0.70544700	1.58941100	-0.00007300
O	3.10507300	0.11802900	-1.28594000
O	1.02002500	-0.66355300	-0.00037600
S	2.72151700	-0.48048700	0.00006400
O	3.10451800	0.11778500	1.28634800
C	-1.21479800	0.15285200	-0.00008300
C	-2.12372400	1.21943500	0.00004400
C	-1.68006400	-1.16763000	-0.00006700
C	-3.48931300	0.96322400	0.00009300

H	-1.74510000	2.23380900	0.00000600
C	-3.04886900	-1.41533300	-0.00002400
H	-0.97456900	-1.98721600	-0.00017000
C	-3.95255500	-0.35313300	0.00008100
H	-4.19240700	1.78780700	0.00014000
H	-3.41054000	-2.43679800	-0.00008400
H	-5.01855500	-0.55068800	0.00012500

TSIM2-P2

C	-0.32863800	0.30837000	0.91567000
H	-0.24495200	0.64470100	2.03050600
O	-0.90886300	1.49011000	0.75259800
O	-1.70641600	0.84734400	-1.01778200
C	1.07781000	0.09051000	0.40508300
C	1.86169100	1.18796200	0.03587000
C	1.60120800	-1.20456500	0.34214300
C	3.15598100	0.98359600	-0.43032000
H	1.44487200	2.18572800	0.09565500
C	2.89831800	-1.40153700	-0.12201300
H	0.98512800	-2.04331100	0.64116200
C	3.67601900	-0.30946400	-0.50750400
H	3.75906600	1.83114000	-0.73453600
H	3.30257000	-2.40533400	-0.18223600
H	4.68710500	-0.46510000	-0.86638300
S	-2.36733300	-0.46029200	-0.61844000
O	-1.15343700	-0.74663700	0.92787200
O	-3.69513100	-0.37986500	-0.02802700

TSIM2-P3

C	0.39285000	0.35672700	-0.29653400
H	0.62030800	1.32137800	-0.76913600
O	0.36527000	0.39166100	1.05897200
O	2.27954200	0.02394500	1.31770500
C	-1.15153900	0.15592300	-0.12176400
C	-1.98136300	1.28597000	-0.11081400
C	-1.67833400	-1.14125900	-0.02502400
C	-3.35812700	1.10909600	-0.09315000
H	-1.54532200	2.27724300	-0.12901400
C	-3.05410000	-1.30117300	-0.00708500
H	-1.00270300	-1.98554600	0.00129500
C	-3.89252300	-0.18036300	-0.04076400
H	-4.01414200	1.97101900	-0.10850000
H	-3.48055200	-2.29608000	0.03799700
H	-4.96796500	-0.31449400	-0.01707100

S	2.84869300	-0.39331900	-0.05485300
O	1.08360800	-0.68410300	-0.80031700
O	3.41534500	0.71975400	-0.82224900

TSIM2-4

C	0.34067600	0.40898900	0.28885400
H	0.61197200	1.47147700	0.29874600
S	2.69954800	-0.23851600	-0.42771300
O	2.51367100	-0.71518000	1.02678900
O	0.61374900	-0.22663900	1.45030700
O	1.07030100	-0.21668200	-0.82706200
O	3.27164300	1.09891200	-0.61822800
C	-1.15129400	0.19867000	0.08182900
C	-2.01367600	1.28684600	0.22490300
C	-1.65055600	-1.07763000	-0.19321000
C	-3.38241300	1.10464700	0.04645400
H	-1.61746300	2.26828000	0.45913800
C	-3.01826400	-1.25052400	-0.37174500
H	-0.96810000	-1.91341400	-0.27733300
C	-3.88409600	-0.16199800	-0.25139800
H	-4.05464000	1.94949000	0.13975500
H	-3.41118300	-2.23454000	-0.59913600
H	-4.95051900	-0.30231500	-0.38631900

IM4

C	-0.21679700	-0.67485500	0.39007200
H	-0.49851900	-1.58941600	-0.14836800
S	-2.72500700	0.17574700	-0.01154600
O	-3.02236800	-1.18746400	-0.48840600
O	-0.24838400	-0.83529800	1.71185000
O	-1.07266200	0.41910400	-0.09491700
O	-3.30640100	1.35939300	-0.65118000
C	1.22344200	-0.26302700	0.09348300
C	2.10951200	-1.18859600	-0.46358900
C	1.65896700	1.01760000	0.45177400
C	3.42595900	-0.81406300	-0.70652900
H	1.76727300	-2.18468900	-0.71955000
C	2.97871200	1.38306100	0.20469300
H	0.96277800	1.71780300	0.89434200
C	3.86135300	0.47039000	-0.37114900
H	4.11196500	-1.52006800	-1.15966000
H	3.31877600	2.37804100	0.46660000
H	4.88946500	0.75744300	-0.55991900

TSIM4-P1

C	-0.24477100	1.30006100	0.45627000
H	-0.85690900	0.96863600	1.33367900
S	-2.37739700	-0.38438100	-0.32632500
O	-2.51844000	-0.31009600	1.15453600
O	-0.19838000	2.55583300	0.35602200
O	-1.34356100	0.72671900	-0.75531700
O	-2.10705700	-1.68757500	-0.96125200
C	0.99259900	0.48265700	0.25943600
C	1.10031900	-0.76720400	0.87879400
C	2.03520900	0.97443100	-0.53568400
C	2.24626400	-1.53215700	0.68817300
H	0.29363600	-1.13890500	1.50122000
C	3.17493500	0.20302200	-0.72173600
H	1.93897500	1.94358900	-1.00997000
C	3.28188100	-1.04832200	-0.11056100
H	2.33049500	-2.50301000	1.16163200
H	3.98007100	0.57302900	-1.34564600
H	4.17297600	-1.64721100	-0.25978500

TSIM4-P4

C	0.12452000	0.74069100	-0.63168300
H	0.48999100	0.19913100	-1.52974500
S	2.94921600	-0.05109300	0.21649800
O	2.98377500	-0.71068900	-1.09161000
O	0.12770700	2.04172900	-0.96212300
O	0.81055300	0.36480900	0.50998800
O	3.34655700	-0.81606500	1.39302200
C	-1.28213100	0.21821000	-0.28175200
C	-1.73351700	-1.00131000	-0.79714700
C	-2.08101300	0.96448800	0.59584600
C	-3.00523300	-1.45253900	-0.46643000
H	-1.09827200	-1.58338800	-1.45440100
C	-3.35244000	0.50463400	0.91788200
H	-1.70680800	1.89243100	1.00939300
C	-3.81517100	-0.70108200	0.38928200
H	-3.36610500	-2.39013600	-0.87272700
H	-3.98051600	1.08289200	1.58527200
H	-4.80457700	-1.06028200	0.64803100

TSIM4-P5

C	-0.17076300	0.65175200	-0.03015500
H	-0.09894500	0.74805900	1.62802600
S	-2.71928600	-0.02638400	-0.12574400

O	-3.09861900	0.26481100	1.26129000
O	-0.54947400	1.77948800	-0.29955200
O	-1.04720700	-0.44256300	-0.11652400
O	-3.32828200	-1.12926000	-0.87660300
C	1.25569000	0.19406000	0.01069800
C	2.25167700	1.16421700	-0.16443100
C	1.60311700	-1.15037200	0.19986200
C	3.58822900	0.78669200	-0.15840700
H	1.96126500	2.19707400	-0.31014200
C	2.94414600	-1.51745600	0.20421500
H	0.82836100	-1.89272500	0.33784800
C	3.93632400	-0.55278400	0.02664900
H	4.35971200	1.53432100	-0.30070800
H	3.21584100	-2.55689700	0.34551700
H	4.98048100	-0.84414600	0.03190100

Cartesian coordinates for the optimized structures at the UBH&HLYP/6-311++G(d,p) level of theory

***anti*-PhCHOO**

C	-1.43364607	0.50924507	0.00000000
H	-1.80549400	1.52522610	0.00000000
O	-2.27628114	-0.39231286	0.00000000
O	-3.60852611	-0.01872577	0.00000000
C	-0.02223309	0.21703597	0.00000000
C	0.87281298	1.28405890	0.00000000
C	0.45664581	-1.09452806	0.00000000
C	2.23380797	1.04587681	0.00000000
H	0.50010206	2.29452193	0.00000000
C	1.81449779	-1.32419816	0.00000000
H	-0.24104925	-1.91282301	0.00000000
C	2.70315687	-0.25625223	0.00000000
H	2.92445603	1.87052275	0.00000000
H	2.18706772	-2.33341119	0.00000000
H	3.76312186	-0.44315731	0.00000000

SO_2

S	0.00000000	0.36542200	0.00000000
O	1.22992000	-0.36553900	0.00000000
O	-1.22992000	-0.36530500	0.00000000

RC1

C	-0.02403000	-0.36869300	0.86833200

H	-0.38232100	-1.36105700	1.08278900
O	-0.77503600	0.59179900	1.14011300
O	-2.06916800	0.22754700	1.49460500
C	1.30118100	-0.13506900	0.37524300
C	2.13572900	-1.23661700	0.19851000
C	1.75398400	1.15079400	0.07157200
C	3.42177500	-1.05528600	-0.26851800
H	1.77110400	-2.22483400	0.42102600
C	3.03761100	1.32278100	-0.39367500
H	1.09403000	1.99035000	0.19691400
C	3.86925400	0.22212900	-0.56176400
H	4.06990500	-1.90191100	-0.40827300
H	3.39499900	2.30860700	-0.63256900
H	4.87098200	0.36415500	-0.92936100
S	-2.92449100	-0.29532400	-0.45109600
O	-1.71901200	-1.03465600	-0.82055100
O	-3.06176600	0.98401400	-1.09556400

TSRC1-1

C	-0.13665800	-0.78292500	0.57471000
H	-0.41930700	-1.80693300	0.41064400
O	-0.88124300	-0.07298800	1.32119000
O	-2.19663100	-0.51950600	1.29640500
C	1.18633600	-0.31547500	0.26590600
C	2.10753800	-1.22884000	-0.23842900
C	1.54509700	1.01857800	0.45811000
C	3.39113100	-0.81611900	-0.53407800
H	1.81445900	-2.25231200	-0.39890700
C	2.82743400	1.42288000	0.16029800
H	0.81446000	1.71794300	0.82281300
C	3.74804600	0.50690900	-0.33205200
H	4.10773900	-1.51732300	-0.92307500
H	3.11297200	2.45014800	0.30146700
H	4.74745400	0.83086100	-0.56668500
S	-2.72604400	-0.03609500	-0.58743500
O	-1.41405100	-0.51796300	-1.10136300
O	-2.82990200	1.40109400	-0.56299300

IM1

C	-0.30649000	-0.89977600	-0.13339900
H	-0.34204700	-1.93947900	-0.44089700
O	-0.91025500	-0.74626000	1.12274100
O	-2.28978200	-0.89172800	0.84760200
C	1.07931300	-0.35716400	-0.07633300

C	2.15491800	-1.20344900	-0.27952800
C	1.28887900	0.98783600	0.19605400
C	3.44719500	-0.70946400	-0.20937600
H	1.98741900	-2.24618700	-0.49153600
C	2.57762400	1.47758600	0.26560700
H	0.44575600	1.63972800	0.34592000
C	3.65693600	0.62977600	0.06285800
H	4.28319800	-1.36787600	-0.36844900
H	2.74330900	2.51982200	0.47571200
H	4.66016900	1.01639200	0.11603400
S	-2.61265100	0.18019000	-0.41319500
O	-1.13228800	-0.15300700	-1.03178200
O	-2.58837800	1.53380500	0.06381800

RC2

C	0.06862900	0.19566800	-0.69179900
H	0.59262100	-0.59272500	-1.20520900
O	0.66874000	1.27979700	-0.52114700
O	2.02183500	1.23163300	-0.81716900
C	-1.30945400	0.06050300	-0.32146100
C	-1.95186000	-1.13713700	-0.62743000
C	-2.00033800	1.08686500	0.32573100
C	-3.28097600	-1.30731900	-0.29622800
H	-1.40595000	-1.92750600	-1.11355300
C	-3.32585900	0.91001300	0.65094800
H	-1.49031800	2.00223000	0.56644500
C	-3.96433100	-0.28443800	0.33972500
H	-3.78069700	-2.23087000	-0.52798700
H	-3.86639400	1.69475500	1.14971200
H	-5.00033300	-0.41707200	0.60015800
S	2.89844000	-0.15860900	0.60321100
O	1.58493800	-0.66989300	0.99408400
O	3.61963400	-0.98353600	-0.33050000

TSRC2-2

C	-0.17090200	0.11142800	0.69330400
H	-0.65754900	-0.69477400	1.21367400
O	-0.74839300	1.24237900	0.68435000
O	-2.11509600	1.11749200	0.85333000
C	1.21437200	0.02439300	0.32048200
C	1.89956000	-1.15205000	0.60973300
C	1.86502300	1.08269900	-0.31260500
C	3.23466200	-1.26771700	0.27858200
H	1.38353800	-1.96914800	1.08423300

C	3.19777900	0.96093600	-0.63820300
H	1.31974800	1.97932500	-0.54569000
C	3.88076800	-0.21138300	-0.34188100
H	3.76833300	-2.17496900	0.49911300
H	3.70769200	1.77196600	-1.12717300
H	4.92122800	-0.30186400	-0.60262700
S	-2.74620500	-0.12317900	-0.63021400
O	-1.34826500	-0.49830200	-0.96503300
O	-3.44215600	-1.10275700	0.16552900

IM2

C	0.32943200	0.43998300	0.23873000
H	0.59212700	1.48857100	0.32760000
O	0.79427100	-0.26753600	1.35167900
O	2.19432800	-0.20819200	1.23220500
C	-1.13016000	0.21464300	0.06658200
C	-1.99572200	1.29447500	0.09165000
C	-1.62443600	-1.07114600	-0.10268000
C	-3.35810300	1.09356400	-0.05360400
H	-1.60894300	2.29088800	0.22376600
C	-2.98322700	-1.26961100	-0.24659500
H	-0.94581600	-1.90512100	-0.12291200
C	-3.85055500	-0.18768000	-0.22192800
H	-4.02946700	1.93411600	-0.03510100
H	-3.36836500	-2.26576200	-0.37794500
H	-4.90925100	-0.34600000	-0.33501200
S	2.63249500	-0.36691500	-0.42239800
O	1.07245600	-0.13111100	-0.84212200
O	3.41724700	0.80541300	-0.68613200
